# Assessment of Knowledge and Attitudes Toward Potential Drug–Drug and Drug–Food Interactions and Associated Factors Among Health Care Professionals at the University of Gondar Comprehensive Specialized Hospital, Northwest Ethiopia: A Cross‐Sectional Study

**DOI:** 10.1002/hsr2.73362

**Published:** 2026-10-08

**Authors:** Melese Legesse Mitku, Yeniewa Kerie Anagaw, Abera Dessie Dagnaw, Derso Teju Geremew, Yeshiwas Ayale Ferede, Tewodros Denekew Haile, Minichil Chanie Worku, Liknaw Workie Limenh, Azmeraw Bekele, Wondim Ayenew

**Affiliations:** ^1^ Department of Pharmaceutical Chemistry School of Pharmacy, College of Medicine and Health Sciences, University of Gondar Gondar Ethiopia; ^2^ Department of Pharmaceutical Chemistry School of Pharmacy, College of Medicine and Health Sciences, Bahir Dar University Bahir Dar Ethiopia; ^3^ Department of Pharmaceutics School of Pharmacy, College of Medicine and Health Sciences, University of Gondar Gondar Ethiopia; ^4^ Department of Reproductive Health Midwifery, Teda Health Sciences College Gondar Ethiopia; ^5^ Department of Social and Administrative Pharmacy School of Pharmacy, College of Medicine and Health Sciences, University of Gondar Gondar Ethiopia; ^6^ Department of Social and Administrative Pharmacy School of Pharmacy, College of Medicine and Health Sciences, Bahir Dar University Bahir Dar Ethiopia

**Keywords:** drug–drug interactions, drug–food interactions, Ethiopia, healthcare professionals, knowledge

## Abstract

**Background and Aims:**

The increased likelihood of potential drug–drug interactions (PDDIs) and food–drug interactions (FDIs) is primarily attributed to the increasing use of multiple medications and the practice of taking medicines with food. Healthcare professionals' (HCPs') knowledge and attitudes toward PDDIs and FDIs play a critical role in ensuring patient safety and effective medication management. An increase in PDDIs and FDIs can compromise patient safety, decrease treatment efficacy, and increase healthcare costs. Therefore, this study aimed to assess the knowledge and attitudes toward PDDIs and FDIs, as well as associated factors, among HCPs at the University of Gondar Comprehensive Specialised Hospital.

**Methods:**

A cross‐sectional study was conducted at the University of Gondar Comprehensive Specialized Hospital. Healthcare professionals' knowledge of PDDIs and FDIs was assessed using a self‐administered questionnaire. To examine knowledge predictors, we conducted a regression analysis. Data analysis was done using SPSS version 27.

**Results:**

There were 328 HCPs involved in this study; 325 completed the study, with a 99.1% response rate; *n* = 3 were excluded due to declining participation or unavailability during data collection. For acetaminophen, codeine, and amoxicillin, the HCPs' PDDIs knowledge levels were 219 (67.4%), whereas 66 (20.3%) were for carbamazepine and cimetidine. Regarding the FDI‐related questions, HCPs answered items most frequently correctly on patients taking sulfa drugs with folate‐containing foods (251; 77.2%), followed by patients taking antibiotics who should avoid acidic foods such as citrus juice (245; 75.4%). Multivariable analysis revealed that being trained as a pharmacists: AOR: 13.222, 95% CI: 6.785–30.769; medical doctors: AOR 11.265, 95% CI: 8.205–14.960; and age > 40 years: AOR 12.673, 95% CI: 1.875–85.646 were significantly associated with HCPs' knowledge of PDDIs and FDIs.

**Conclusion:**

Based on the results of this study, a minority of HCPs demonstrated good knowledge of potential drug–drug interactions (30.5%) and food–drug interactions (32.0%), indicating notable gaps in knowledge.

## 1 Background

Food and medication are essential elements of patients' treatment and well‐being [1, 2]. Diseases can be both prevented and treated with various foods and drugs, which is why patients are often advised to consume healthy foods. However, due to potential interactions between food products and medications, individuals should be cautious about using food and drugs simultaneously [3].

Drug–drug and drug–food interactions can lead to an increase in toxicity or a decrease in efficacy. These can occur with both prescription‐only and over‐the‐counter drugs [4, 5]. The mechanisms behind these interactions can be categorized as pharmaceutical, pharmacokinetic, or pharmacodynamic [6]. Pharmacokinetic interactions arise when processes related to drug absorption, distribution, metabolism, and excretion are altered by the concurrent use of food and medicine [7]. However, pharmacodynamic interactions occur when a drug's clinical effect is altered by food or another medication. These interactions may produce antagonistic, synergistic, or additive effects [8].

In recent years, the use of multiple medications and drugs with food has become widespread worldwide [9]. Multiple medications are commonly associated with an increased risk of potential drug–drug interactions (PDDIs) [10, 11] and drug–food interactions (FDIs) [10]. The prevalence of both PDDIs [11, 12, 13, 14] and FDIs is high globally [12, 13, 14], contributing to complex adverse drug effects (ADEs) [1, 15, 16]. ADEs may affect therapeutic effects, including treatment outcomes, adherence, and toxicity of the drugs [17, 18]. These effects lead to increased hospitalization and healthcare costs, compromise quality of life [19], and can even cause death [20]. The prevalence of PDDIs is reported to be as high as 74.41% [21], while the prevalence of potential FDIs varies from 6% to 70% across different countries [22]. Around 2.8% of hospital admissions are caused by ADEs related to PDDIs [11]; this may result in negative consequences such as diminished effectiveness, an increase in adverse responses, worsening of the disease, and death [20]. One study found that patients with severe DDIs were three times more likely to die than those with moderate interactions [23]. Moreover, exposure to DDIs can lead to unnecessary healthcare costs, with preventable ADRs costing between €2851 and €9015 in the USA and Europe [24]. Research has shown that reducing DDIs by just 1% could result in annual savings of $29,250 in indirect costs [25].

The evaluation of healthcare professionals' (HCPs) knowledge, attitudes, and practices (KAP) regarding life‐threatening DDIs and the implementation of specific interventions can enhance patient safety [26]. KAP related to PDDIs is essential for developing and implementing strategies to reduce their occurrence. For example, in the United States, the economic burden of drug‐related events decreased from US$ 177.4 billion in 2001 to US$ 21 billion in 2014, largely due to the implementation of effective strategies to prevent PDDIs [27]. Similarly, in Europe, a 40% reduction in PDDIs has been reported due to successful strategies for reporting and resolving these interactions [28]. The KAP of HCPs plays a critical role in reducing DDI‐related complications and hospital stays [29]. Moreover, adequate KAP about drug–food interactions can help minimize the harmful effects of such interactions in patients [30].

Currently, PDDIs and FDIs do not receive adequate attention from HCPs, despite their potential to cause significant health hazards [31, 32, 33]. Numerous studies have assessed the inability of HCPs to recognize suspected PDDIs and FDIs [1, 34]. Pharmacy staff are responsible for restricting the use of dangerous prescription regimens to prevent dispensing combination medications that could cause major PDDIs [11, 29]. Beyond dispensing medications and reviewing prescriptions, they are also responsible for patient counseling [35, 36, 37]. HCPs must thus comprehend PDDIs and FDIs to properly educate patients, avoid major side effects, and improve the efficacy of treatment [38].

In high‐income countries, systems are routinely implemented to identify and mitigate PDDIs and FDIs. In contrast, in low‐resource settings such as Ethiopia, the infrastructure required to detect and prevent these interactions is often limited. Consequently, HCPs must rely largely on their own knowledge and clinical judgment when prescribing and dispensing medications. To the best of our knowledge, no previous study has assessed the KAP of healthcare professionals regarding PDDIs and FDIs in the study area. Therefore, this study aims to explore the knowledge and attitudes of HCPs regarding the importance of considering potential drug‐drug and drug–food interactions in clinical practice. It also seeks to identify any gaps in knowledge or misconceptions among HCPs at the University of Gondar Comprehensive and Specialized Hospital. Ultimately, this study is needed in the study area to provide baseline data for designing, developing, and implementing targeted clinical strategies to mitigate PDDIs and FDIs.

## 2 Methods

### 2.1 Study Settings

In this study, a cross‐sectional study was conducted to assess HCPs at the University of Gondar Comprehensive Specialized Hospital, Gondar, Ethiopia, over 3 months from January to March 2024. Gondar is located 727 km from Addis Ababa, the capital city of Ethiopia. In the city, there is a single government referral hospital affiliated with one of the country's main health science colleges. Additionally, 2 private hospitals and 8 governmental health centers are present in the city.

### 2.2 Study Population

The source population of our study consists of all healthcare professionals working at UOG Specialized and Comprehensive Hospital. However, the study population included full‐time HCPs (pharmacists, medical doctors, medical nurses, and health officers) employed at the University of Gondar Comprehensive Specialized Hospital, with a focus on those involved in prescribing and dispensing medications.

### 2.3 Inclusion and Exclusion Criteria

#### 2.3.1 Inclusion Criteria

We included HCPs who are working at the University of Gondar Comprehensive Specialized Hospital. Those directly involved in patient care and medication management. Professionals with at least 6 months of work experience at the hospital. Individuals who volunteer to participate and provide informed consent.

#### 2.3.2 Exclusion Criteria

The study excluded HCPs who were on leave or unavailable during data collection, those not directly involved in prescribing, dispensing, or administering medications, and those with less than 6 months of work experience. Individuals who decline participation or do not provide informed consent. Professionals with less than 6 months of work experience, as summarized in Figure 1.

**Figure 1:**
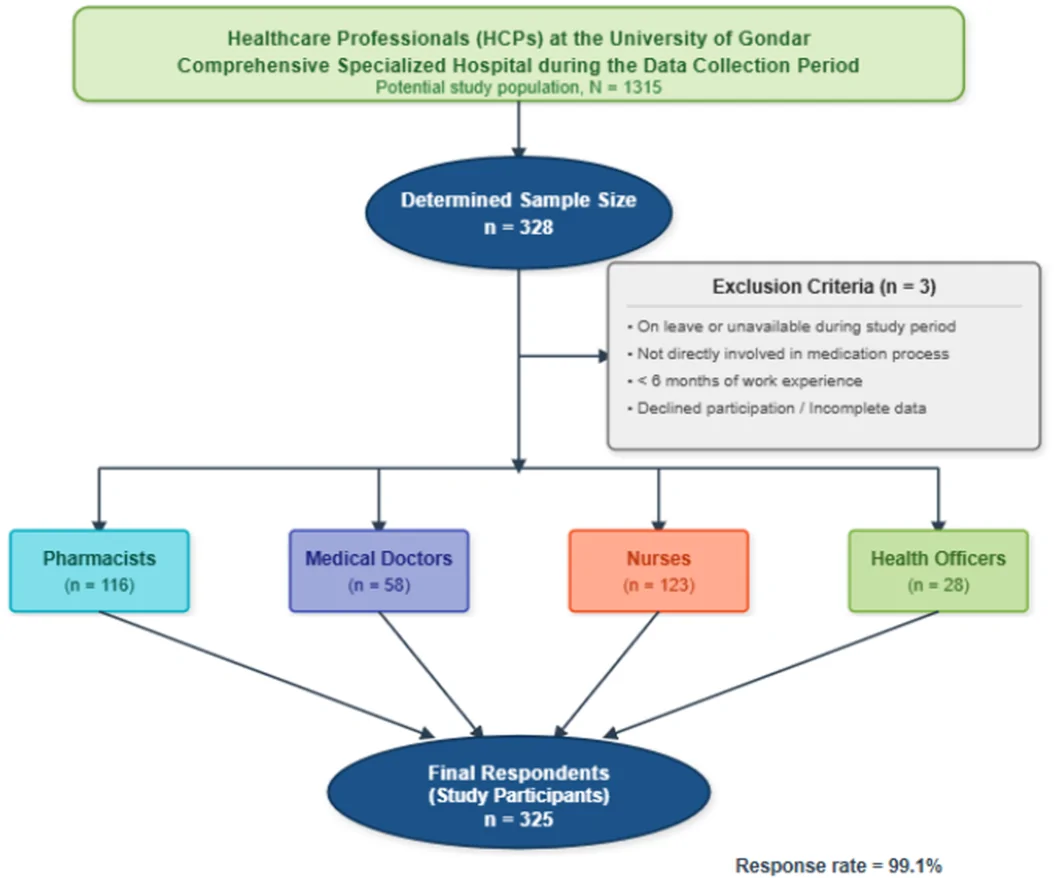
The sampling process was used to select healthcare professionals at the University of Gondar Comprehensive Specialized Hospitals.

### 2.4 Sample Size Determination and Sampling Technique

The single population proportion formula with a 95% confidence level and a 5% margin of error was used to determine the number of participants included in this study [39]. Moreover, a stratified random sampling technique was employed to select the study participants. According to a 2024 hospital human resources report, the source population of our study was estimated to be 1315 full‐time employees of HCPs [40], and the sample was adjusted to 328 using the sample calculation formula. The sample size was estimated as follows:

$$n=\frac{{\left(z\propto /2\right)}^{2}.p.(1-p)}{{\left(d\right)}^{2}}=\frac{{\left(1.96\right)}^{2}.0.5.(1-0.5)}{{\left(0.05\right)}^{2}}=384$$

Were, *n* = Sample size, Z α/2 = Critical value = 1.96, p = Percentage of estimated prevalence = 0.50, d = Precision (margin of error) = 0.05. Since the sample was drawn from a total population of 1315 HCPs (less than 10,000), the corrected sample size was calculated using:

$$\text{Corrected estimated sample size}\;\frac{384\times 1315}{384+1315}=298$$

To account for potential nonresponse and incomplete data, a 10% increase will be added, making the final adjusted estimated sample size 298 + (298 × 0.1) = 328 respondents. Finally, the sample for each healthcare professional was taken based on their respective ratio.

### 2.5 Data Collection Tool and Data Management

A structured self‐administered questionnaire was prepared and used from previously published literature [11, 38, 41, 42, 43]. The questionnaire was divided into five sections. Section 1 collected the sociodemographic data of the HCPs. Section 2 consisted of questions regarding HCPs' history of encountering drug interactions and workload. In Section 3, which was composed of the assessment of the knowledge of life‐threatening PDDIs of particular medication pairings among HCPs, sixteen locally accessible and frequently prescribed medication pairings were included. PDDIs were identified by using the Drug‐Reax® System, which is interactive drug interaction software created by Thomson Micromedex Drug Interactions (Thomson Reuters Healthcare Inc., Greenwood Village, CO, USA) from the US database [44] and Drug.com (https://www.drugs.com/). Among the sixteen drug combinations tested, seven were contraindicated, five could be administered with supervision, and four had no known interactions. Participants were asked to categorize each drug combination into one of four categories without the use of any references: (a) contraindicated; (b) may be used together with monitoring; (c) no interaction; and (d) not sure. To avoid speculation, the “not sure” option was included. Moreover, Section 4 evaluated participants' knowledge of potential drug–food interactions using a 13‐item tool consisting of 44 sub‐items. For each item, participants were requested to select a binary response (‘Yes’ or ‘No’). The knowledge levels were classified according to Bloom's cut‐off points [26]. The final section of the questionnaire assessed the attitudes of HCPs toward clinically significant and life‐threatening drug‐drug and drug–food interactions.

To ensure the quality of the data collection tool, a pilot study was performed. The participants in the pilot study were excluded from the study. Following the pilot study, ambiguous or unclear items were revised, repetitive questions were removed, and the wording and ordering of selected items were adjusted to improve comprehensibility and consistency. Throughout the data management, storage, and analysis processes, the consistency and completeness of each completed questionnaire were checked.

### 2.6 Data Collection and Statistical Analysis

The data was collected by four trained undergraduate pharmacy students. Completed questionnaires were checked for completeness before data entry. All collected questionnaires were complete, with no missing data observed. Consequently, all records were included in the final analysis.

### 2.7 Measuring Tools

Knowledge: Each knowledge‐related question was categorized into four categories: coded as no interaction = 1, contraindicated = 2, may be used together with monitoring = 3, and not sure = 4, then recoded as correct answer = 1; incorrect answer = 0. After each respondent's response was summed, his or her level of knowledge was categorized as poor, moderate, or good, based on the Bloom cut‐off point. Those who scored between 80% and 100% (12–14 points) were considered to have “good knowledge”, while those who scored between 60% and 79% (9–11 points) were considered to have “moderate knowledge”. On the other hand, HCPs who scored below 60% (0–8 points) were classified as having “poor knowledge”. Moreover, Participants' drug–food interaction knowledge was categorized using Bloom's cut‐off points into poor (< 60%; 0–26 points), moderate (60%–79%; 27–34 points), and good knowledge (80%–100%; 35–44 points) based on their total score across 44 sub‐items. For both PDDI and FDI, those with moderate knowledge were classified as having good knowledge for the sake of regression. Responses to attitude questions were categorized into two categories. The answer “yes” was scored 1, while the answer “no” was scored 0 [26, 45]. The mean of each attitude‐related question and the grand mean were computed. Then, categorization was performed based on the grand mean score. Those who scored above the grand mean were considered to have a “positive attitude”, while those who scored below the grand mean were considered to have a “negative attitude” [45, 46, 47].

The collected data were analyzed using the Statistical Package for Social Sciences (SPSS), version 27.0 (SPSS, Chicago, IL, USA). Means, frequency distributions, and descriptive statistics were used. The presence and degree of association between HCPs' PDDI/FDI knowledge and each independent variable were evaluated using bivariate logistic regression analysis. Next, a multivariable logistic regression model was used to account for any potential confounding factors. First, we performed bivariate binary logistic regression to identify candidate variables for the final analysis using a *p*‐value < 0.25 as the cut‐off point [48]. Then, multivariable logistic regression was carried out to determine statistically significant variables related to HCPs' PDDI/FDI knowledge at a *p*‐value < 0.05.

The goodness‐of‐fit of the logistic regression model was assessed using the Hosmer–Lemeshow test, which indicated an adequate model fit (*χ* ^2^ = 7.4, *p* = 0.49). Multicollinearity among the independent variables was evaluated using variance inflation factors, which ranged from 1.2 to 2.4, indicating no evidence of problematic multicollinearity. The internal consistency reliability of the questionnaire was assessed using Cronbach's alpha, yielding a coefficient of 0.80, which demonstrated good reliability.

## 3 Results

### 3.1 Socio‐Demographic Characteristics of the Respondents

A total of 328 HCPs were invited, of whom 325 participated in the study, for a response rate of 99.1%. The majority of respondents were males (53.2%). The respondents' ages ranged from 24 to 60 years, with an average age of 31.96 ± 7.22 (mean ± SD). Most of the respondents, 123 (37.9%), were in the nursing profession. About 150 (46.2%) of the respondents were under 30 years old. The majority of participants 176 (54.2%), 167 (51.4%), and 222 (68.3%) had degrees and above, had less than 5 years of working experience, and had not received training regarding drug–drug or drug–food interactions, respectively (Table 1).

**Table 1:** Socio‐demographic characteristics of healthcare professionals at UoG‐CSH, Northwest Ethiopia, 2024.

Variables	Categories	Frequency	Percent
Sex	Male	173	53.2
	Female	152	46.8
Health care professionals	Pharmacist	116	35.7
	Medical doctors	58	17.9
	Nurse	123	37.9
	Health officer	28	8.6
Age	< 30 years	150	46.2
	30–40 years	132	40.6
	> 40 years	43	13.2
Education level	Diploma	149	45.8
	Degree and above	176	54.2
Work experience	< 5 years	167	51.4
	6–10 years	84	25.8
	> 10 years	74	22.8
Received training in drug interactions	Yes	103	31.7
	No	222	68.3
Were drug interactions part of the undergraduate course	Yes	266	81.9
	No	59	18.2

### 3.2 HCPs' Knowledge of PDDIs

From the 16 drug pairs, the average percentages of respondents who correctly identified ranged from 20.3% to 67.4% for the drug combinations of carbamazepine and cimetidine (which may be used together with monitoring) and the drug combinations of acetaminophen/codeine and amoxicillin (with no interaction), respectively. In this study, of 16 drug pairs, only six were accurately classified by half of the participants. Of the 7 contraindicated drug pairs, only 3, nitroglycerin and sildenafil, clopidogrel and erythromycin, and warfarin and cimetidine, were properly identified by 172 (53.8%), 172 (52.9%), and 105 (52.2%) of the respondents, respectively. Among 16 drug combinations, 5 drug pairs were correctly identified by less than one‐third of participants (Table 2). Overall, Figure 2 shows that 30.5% and 32.0% of HCPs exhibited a good level of knowledge regarding PDDIs and DFIs, respectively.

**Table 2:** Knowledge of healthcare professionals regarding PDDIs.

Drug pairs	No interaction	Should not be used together (contraindicated)	May be used together with monitoring	Not sure
Acetaminophen/codeine and amoxicillin	**219 (67.4%)**	53 (16.3%)	41 (12.6%)	12 (3.7%)
Warfarin and cotrimoxazole	86 (26.5%)	130 (40.0%)	**86 (26.5%)**	23 (7.1%)
Warfarin and digoxin	**111 (34.2%)**	108 (33.2%)	72 (22.2%)	34 (10.5%)
Methotrexate and cotrimoxazole	54 (16.6%)	**46 (14.2%)**	181 (55.7%)	44 (13.5%)
Digoxin and sildenafil	**83 (25.5%)**	149 (45.8%)	53 (16.3%)	40 (12.3%)
Simvastatin and itraconazole	178 (54.8%)	**67 (20.6%)**	56 (17.2%)	24 (7.4%)
Nitroglycerin and sildenafil	41 (12.6%)	**175 (53.8%)**	46 (14.2%)	63 (19.4%)
Theophylline and ciprofloxacin	71 (21.8%)	70 (21.5%)	**130 (40.0%)**	54 (16.6%)
Carbamazepine and cimetidine	136 (41.8%)	90 (27.7%)	**66 (20.3%)**	33 (10.2%)
Clopidogrel and Erythromycin	80 (24.6%)	**105 (32.3%)**	86 (26.5%)	54 (16.6%)
Simvastatin and Clarithromycin	139 (42.8%)	**72 (22.2%)**	45 (13.8%)	69 (21.2%)
Allopurinol and Pyrazinamide	**132 (40.6%)**	72 (22.2%)	57 (17.5%)	64 (19.7%)
Praziquantel and Rifampicin	85 (26.2%)	**39 (12.0%)**	166 (51.0%)	35 (10.8%)
Warfarin and cimetidine	74 (22.8%)	**172 (52.9%)**	55 (16.9%)	24 (7.4%)
Warfarin and itraconazole	76 (23.2%)	39 (12.0%)	**136 (41.8%)**	74 (22.8%)
Metformin and erythromycin	**129 (39.7%)**	50 (15.4%)	72 (22.2%)	74 (22.8%)

*Note:* bolded values indicate the correct responses.

**Figure 2:**
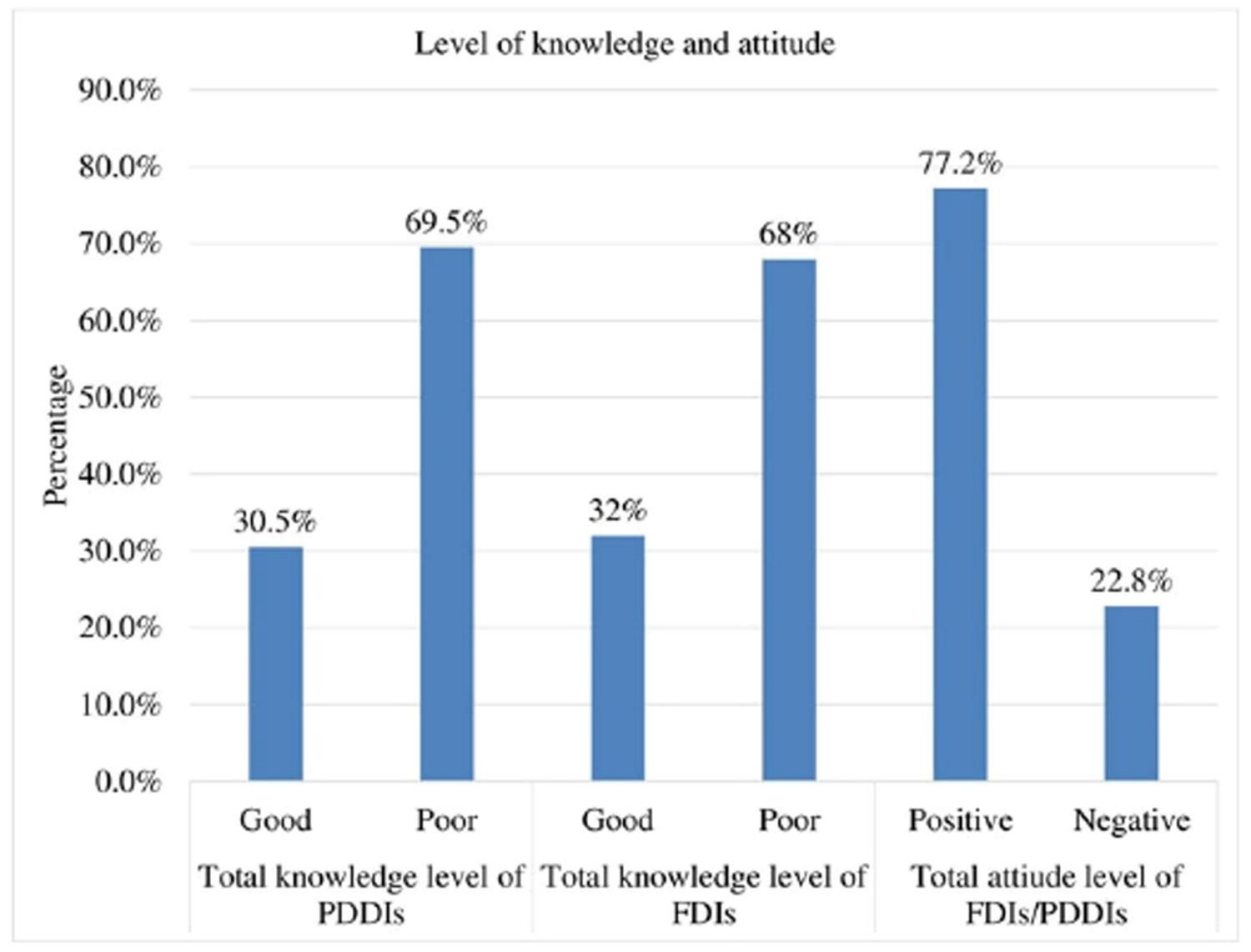
Healthcare professionals' level of knowledge and attitudes towards FDIs/PDDIs.

### 3.3 HCPs' Knowledge of Food–Drug Interactions

In this study, HCPs demonstrated a high knowledge level for some individual questions regarding food–drug interactions. According to the knowledge level, 86.8% and 77.2% of participants knew that sulfa drugs interact with vitamin B12 and folate, respectively. Three‐fourths of participants responded that patients taking antibiotics like tetracycline and fluoroquinolones should avoid iron‐rich foods, as well as avoid alcohol while taking metronidazole. Moreover, participants answered correctly that patients taking antibiotics such as tetracycline or fluoroquinolones and monoamine oxidase inhibitors should avoid meat (68.0%) and fava beans (64.0%), respectively.

The study revealed that 59.7% and 53.2% of participants answered correctly that potassium‐sparing diuretics should be taken with minerals and antacids, respectively. In contrast, fewer than half (43.4%, 48.0%, 48.9%, and 39.7%) of the participants were aware of the interactions between monoamine oxidase inhibitors and cheese, fresh meats, wine, and fermented products, respectively, as shown in Table 3. In general, HCPs have less than one‐third (32%) of knowledge about FDIs, as illustrated in Figure 2.

**Table 3:** Knowledge of healthcare professionals regarding FDIs.

Knowledge items and sub‐items	Correct response	Yes, *n* (%)	No, *n* (%)
Q1. A patient taking theophylline should avoid consuming large quantities of:			
Tea	Yes	53 (16.3%)	272 (83.7%)
Coffee	Yes	153 (47.1%)	172 (52.9%)
Chocolate	Yes	142 (43.7%)	183 (56.3%)
Q2. A patient taking antibiotics such as tetracycline and fluoroquinolones should avoid:			
Milk	Yes	190 (58.5%)	135 (41.5%)
Dairy products	Yes	189 (58.2%)	136 (41.8%)
Iron‐rich food	Yes	245 (75.4%)	80 (24.6%)
Meat	No	221 (68.0%)	104 (32.0%)
Q3. A patient on monoamine oxidase inhibitors should avoid:			
Cheese	Yes	141 (43.4%)	184 (56.6%)
Fresh meats	No	156 (48.0%)	169 (52.0%)
Wine	Yes	159 (48.9%)	166 (51.1%)
Fava beans	Yes	208 (64.0%)	117 (36.0%)
Fermented products	Yes	129 (39.7%)	196 (60.3%)
Q4. A patient taking antibiotics should avoid acidic foods such as:			
Tomato sauce	Yes	142 (43.7%)	183 (56.3%)
Potato	No	98 (30.2%)	227 (69.8%)
Coffee	Yes	115 (35.4%)	210 (64.6%)
Citrus juices	Yes	245 (75.4%)	80 (24.6%)
Q5. Caffeine increases the risk of toxicity of the following drugs:			
Pseudoephedrine	Yes	127 (39.1%)	198 (60.9%)
Theophylline	Yes	110 (33.8%)	215 (66.2%)
Quinidine	Yes	103 (31.7%)	222 (68.3%)
Diazepam	No	80 (24.6%)	245 (75.4%)
Q6. The following drugs should be taken with a high‐fat diet:			
Griseofulvin	Yes	103 (31.7%)	222 (68.3%)
Albendazole	Yes	219 (67.4%)	106 (32.6%)
Esomeprazole	No	86 (26.5%)	239 (73.5%)
Q7. A patient should avoid alcohol with the following drugs:			
Metronidazole	Yes	240 (73.8%)	85 (26.2%)
Diazepam	Yes	188 (57.8%)	137 (42.2%)
Antihistamines	Yes	194 (59.7%)	131 (40.3%)
Metformin	Yes	190 (58.5%)	135 (41.5%)
Isoniazid	Yes	173 (53.2%)	152 (46.8%)
Q8. A patient should avoid taking antibiotics such as Sulfa drugs with:			
Vitamin B12	No	282 (86.8%)	43 (13.2%)
Folate	Yes	251 (77.2%)	74 (22.8%)
Q9. The following drugs should not be taken with a high‐protein diet:			
Warfarin	No	135 (41.5%)	190 (58.5%)
Propranolol	No	135 (41.5%)	190 (58.5%)
Cimetidine	No	129 (39.7%)	196 (60.3%)
Q10. The following drugs should not be taken with salty foods:			
Oral contraceptives	No	85 (26.4%)	240 (73.8%)
Steroids	Yes	137 (42.2%)	188 (57.8%)
Q11. The following drugs should be taken with minerals:			
Potassium‐sparing diuretics	No	194 (59.7%)	131 (40.3%)
Tetracycline	No	66 (20.3%)	259 (79.7%)
Antacids	No	173 (53.2%)	152 (46.8%)
Q12. Which ART drugs must be taken on an empty stomach:			
Lopinavir/Ritonavir	No	43 (13.2%)	282 (86.8%)
Didanosine and Indinavir	Yes	74 (22.8%)	251 (77.2%)
Zidovudine	No	80 (24.6%)	245 (75.4%)
Q13. Which of the following medications should be taken 1 h after eating food:			
Omeprazole	No	103 (31.7%)	222 (68.3%)
Ranitidine	No	201 (61.8%)	124 (38.2%)
Antihistamines	No	111 (34.2%)	214 (65.8%)

*Note:* Percentage totals reflect participant response proportions (*N* = 325). Typographical percentage errors in original raw counts have been corrected. The assessment instrument consists of 13 main conceptual questions (Q1–Q13). Each conceptual question comprises multiple specific food or drug sub‐items evaluated independently using a binary (Yes/No) format, yielding a total of 44 evaluated sub‐items.

### 3.4 Attitudes of HCPs Towards PDDIs and FDIs

Table 4 summarizes the eight attitude questions about PDDIs and FDIs. The majority of respondents had positive attitudes towards PDDIs and FDIs. More than ninety percent of the respondents responded positively to the following questions: Do you think HCPs are responsible for reporting drug‐drug/food interactions (312, 96.0%); Do you believe that it is important to check drug‐drug/food interactions (303, 93.2%); Have you undergone any drug‐drug/food interaction/adverse drug reaction training program (301, 92.6%); and are you interested in attending any training on drug–drug/food interactions (294, 90.5%). The attitudes towards potential DDIs are summarized in Figure 2.

**Table 4:** Healthcare professionals' attitudes towards PDDIs and FDIs.

Statement	Yes	No	Mean	Attitude
Q1: Do you consider drug–drug/food interaction to be an important Aspect?	269 (82.8%)	56 (17.2%)	0.83	Negative
Q2: In your practice, have you come across any serious drug‐drug/food interaction?	274 (84.3%)	51 (15.7%)	0.84	Negative
Q3: Have you undergone any drug‐drug/food interaction/adverse drug reaction training program?	301 (92.6%)	24 (7.4%)	0.93	Positive
Q4: Are you interested in attending any training on drug‐drug/food interaction	294 (90.5%)	31 (9.5%)	0.90	Positive
Q5: Are you responsible for catching drug‐drug/food interactions	279 (85.8%)	46 (14.2%)	0.86	Negative
Q6: Are you well informed/knowledgeable about drug‐drug/food interactions	218 (67.1%)	107 (32.9%)	0.67	Negative
Q7: Do you believe that it is important to check drug–drug/food interactions	303 (93.2%)	22 (6.8%)	0.93	Positive
Q8: Do you think healthcare professionals are responsible for reporting drug–drug/food reactions	312 (96.0%)	13 (4.0%)	0.96	Positive
Grand mean			**0.865**	

*Note:* Attitude was classified using the grand mean cutoff method. Items with a mean score at or above the overall grand mean (0.865) were classified as a ‘Positive’ attitude, and those below the grand mean as ‘Negative,’ regardless of the absolute proportion of ‘Yes’ responses.

### 3.5 Factors Associated With Knowledge of HCPs Towards PDDIs and FDIs

Sex, health care profession, age, educational level, years of experience, training regarding FDIs, course, and how often FDIs were identified as candidate variables for multivariable logistic regression (*p* value < 0.25) (Table 5). Multivariable analysis revealed that being a pharmacist or medical doctor was associated with FDI knowledge level (AOR: 13.222, 95% CI: 6.785–30.769, AOR: 11.265, 95% CI: 8.205–14.960, respectively). Participants had more than 40 years of age, degree and above educational level, and training on FDIs associated with FDIs' ability to attain a greater knowledge level (AOR: 12.673, 95% CI: 1.875–85.646, AOR: 5.711, 95% CI: 1.954–16.688, AOR: 20.282, 95% CI: 7.098–57.959, respectively).

**Table 5:** Factors associated with knowledge of HCPs toward PDDIs & FDIs.

Variables	Categories	Knowledge level towards PDDI/FDI					
		Good *n* [%]	Poor *n* [%]	COR (95%CI)	AOR (95%CI)		*p* value
Sex	Male	41 (23.7)	132 (76.3)	1	1		0.645
	Female	63 (41.4)	89 (58.6)	2.279 (1.415–3.670)	1.204 (0.546–2.658)		
HCPs	Pharmacist	46 (39.7)	70 (60.3)	1.971 (0.776–5.011)	13.222 (6.785–30.769)		0.013*
	Medical Doctors	20 (34.5)	38 (65.5)	1.950 (1.124–3.385)	11.265 (8.205–14.960)		0.014*
	Nurse	31 (25.2)	92 (74.8)	1.249 (0.647–2.408)	1.010 (0.354–2.882)		0.985
	Health officer	7 (25.0)	21 (75.0)	1	1		
Age group	< 30 yrs	73 (48.7)	77 (51.3)	1	1		
	30–40 yrs	23 (17.4)	109 (82.6)	4.497 (2.587–7.803)	2.272 (0.756–6.825)		0.144
	> 40 yrs	8 (18.6)	35 (81.4)	4.148 (1.805–9.532)	12.673 (1.875–85.646)		0.009*
Educational level	Diploma	72 (48.3)	77 (51.7)	1	1		
	Degree and above	32 (18.2)	144 (81.8)	4.208 (2.552–6.937)	5.711 (1.954–16.688)		0.001*
Work experience in years	< 5 yrs	78 (46.7)	89 (53.3)	1	1		
	6–10 yrs	10 (11.9)	74 (88.1)	3.177 (1.690–5.974)	1.341 (1.069–1.683)		0.011*
	> 10 yrs	16 (21.6)	58 (78.4)	6.485 (3.135–13.147)	11.027 (3.312–36.710)		< 0.001*
Training on FDIs	Yes	66 (64.1)	37 (35.9)	8.637 (5.069–14.717)	20.282 (7.098–57.959)		< 0.001*
	No	38 (17.1)	184 (82.9)	1	1		
The average number of hours spent at work/week	< 48	46 (41.4)	65 (58.6)	1	1		
	48–64	41 (31.8)	88 (68.2)	1.519 (0.895–2.578)	0.674 (0.669–1.815)		0.573
	> 64	17 (20.0)	68 (80.0)	2.183 (1.475–5.433)	0.922 (0.235–3.613)		0.907
Course	Yes	92 (34.6)	174 (65.4)	2.071 (1.047–4.098)	8.649 (1.773–42.192)		0.008*
	No	12 (20.3)	47 (79.7)	1	1		
How often encounter FDIs	Once	72 (43.4)	94 (56.6)	1	1		
	Twice	13 (46.4)	15 (53.6)	0.884 (0.396–1.974)	4.644 (1.164–18.529)		0.030*
	Several times	19 (14.5)	112 (85.5)	4.551 (4.541–8.024)	44.365 (11.119–177.024)		< 0.001*
Number of requests	< 100	81 (40.9)	117 (59.1)	1	1		
	100–200	6 (16.7)	30 (83.3)	3.462 (1.378–8.696)	0.974 (0.966–1.387)		0.560
	> 200	17 (18.7)	74 (81.3)	3.014 (1.656–5.483)	0.553 (0.527–2.399)		0.640

* = Significance level

## 4 Discussion

In this study, the majority of respondents were males (53.2%). A similar study done on the awareness of physicians in DDIs in Addis Ababa reported that 79.3% of the respondents were males [49]. In contrast, in a study done on healthcare providers regarding life‐threatening drug–drug interactions in Penang General Hospital, Malaysia, 64.8% of the participants were female [26]. This study revealed that most of the HCPs were nurse professionals (46.2%). This finding is supported by a study reported by Betelhem Endal et al. at Ayder Mekelle; most of the HCPs were nurse professionals (73.7%) [50], and most of the HCPs were under 30 years of age.

The findings of this study have important implications for medication safety and are closely aligned with the WHO Global Patient Safety Challenge: Medication Without Harm, which emphasizes the prevention of avoidable medication‐related harm through safer prescribing, dispensing, and monitoring practices [51]. According to this research, certain harmful PDDIs were undetected by HCPs. Out of 16 drug pairs, only six were correctly classified by participants more than 50% of the time. Of these, 3, 1, and 2 drug pairs were correctly classified as contraindicated, use with monitoring, and no‐interaction drug pairs, respectively. In general, only 30.5% (95% CI: 25.8–35.4) of HCPs demonstrated a good level of knowledge on PDDI‐related questions, indicating that the majority had moderate or poor knowledge. This finding is in line with studies reported by Albalawi et al. in 2019 in Saudi Arabia [52], Ko et al. in 2008 in the U.S [38]., and Abdo et al. in 2020 [26] in Malaysia. Additionally, most HCPs had low knowledge levels for the majority of food–drug interaction‐related questions. For instance, the interactions between theophylline and tea, coffee, and chocolate were answered correctly by 16.3%, 47.1%, and 43.7% of participants, respectively. Only 39.1%, 33.84%, 31.7%, and 24.6% of participants correctly answered that caffeine increases the toxic effects of pseudoephedrine, theophylline, quinidine, and diazepam, respectively. In general, only 32.0% of HCPs demonstrated a good level of knowledge on FDI‐related questions, indicating that the majority had moderate or poor knowledge. The observed low level of knowledge may result from several factors, including inadequate training, limited awareness of specific interactions, heavy workloads, and insufficient access to resources. However, this finding is in line with previous studies reported by Degefu et al. in 2022 in Ethiopia [53], Alhubail et al. in 2023 in the eastern region of Saudi Arabia [54], and Elshenawi and Elazeem in 2020 in Alexandria, Egypt [55]. Consequently, HCPs with a good knowledge of PDDIs and FDIs are crucial for minimizing their associated risks [56].

The majority of HCPs had positive attitudes towards PDDIs and FDIs. For each attitude‐related question, 77.20% of the participants had an overall positive attitude toward PDDIs and FDIs. This finding is in line with the findings reported by Albalawi et al. 2019 [52].

The results of this study revealed that age greater than 40 years and training in drug interactions were significantly associated with HCPs' knowledge of PDDIs and FDIs. Frequent encounters with PDDIs/FDIs were associated with a higher likelihood of having good knowledge compared with encountering PDDIs/FDIs only once (AOR = 44.365, 95% CI: 11.119–177.024). Additionally, HCPs who participated in training in drug interactions had greater levels of knowledge (AOR: 20.282, 95% CI: 7.098–57.959) than those who did not participate in training. HCPs aged greater than 40 years had greater knowledge (AOR: 12.673, 95% CI: 1.875–85.646) and more training in drug interactions.

Working experience was another factor associated with HCPs' knowledge of FDI/PDDIs, along with how often they encounter and train on PDDIs/FDIs. HCPs who had greater than 10 years of working experience had greater knowledge levels (AOR: 11.027, 95% CI: 3.312–36.710) than those who reported having less than 5 years of experience. This finding is supported by a study conducted by Mahmoud Saeed Abdo et al. in 2020 [57]. Those who participated in drug interactions as part of their undergraduate/postgraduate course studies were more likely to achieve a high knowledge level for FDIs (AOR: 8.649, 95% CI: 1.773–42.192) than those who did not. The findings from the present study also showed that HCPs with a degree and above had greater knowledge (AOR: 5.711, 95% CI: 1.954–16.688) of FDI/PDDI than those with a diploma. This result is supported by a study reported by Osuala EC, Tlou B, and Ojewole EB in 2021 [30]. Compared with nurse professionals, pharmacists, and medical doctors also had higher knowledge levels (AOR: 13.222, 95% CI: 6.785–30.769, AOR: 11.265, 95% CI: 8.205–14.960).

The findings of this study support the need for system‐level strategies to improve the detection of drug–drug and drug–food interactions in routine practice. Therefore, digital clinical decision‐support tools, such as interaction‐checking software, mobile applications, and prescribing alert systems, may help healthcare professionals identify clinically important interactions at the point of care [58, 59]. Although full electronic prescribing systems may not be easily implemented in resource‐limited settings, lower‐cost alternatives such as offline databases, reference charts, and pharmacist‐supported screening systems may be feasible. Moreover, the strong relationship between training and knowledge highlights the importance of continuous professional education, including in‐service training, refresher courses, orientation programs, and multidisciplinary medication safety teaching sessions. Expanding clinical pharmacy services and access to drug information resources may also improve medication safety practices [60, 61].

While this study addresses a critical knowledge gap, the purpose of regression analysis, participants with moderate knowledge were grouped together with those having good knowledge. This classification may introduce bias by potentially overestimating the true competency level of the population. In addition, such grouping may weaken the strength of associations observed in the regression analysis. Moreover, a few of the adjusted odds ratios observed in our analysis were relatively high, which may have produced unstable estimates. The cross‐sectional nature of this study limits causal inference. In addition, because the study was conducted at a single institution, the findings may not be generalizable to all healthcare professionals.

## 5 Conclusion

Based on the results of this study, a minority of HCPs demonstrated good knowledge regarding potential drug–drug interactions (30.5%) and food–drug interactions (32.0%), indicating notable gaps in knowledge. In this study, training on drug‐drug and drug‐food interactions, undergraduate/postgraduate course studies on these interactions, and years of experience were significantly associated with HCPs knowledge of PDDIs and FDIs. Therefore, it is advised that HCPs should receive additional course studies and training about these interactions to improve their comprehension of these interactions. However, a few of the adjusted odds ratios observed in our analysis were relatively high, possibly due to small numbers in some categories, which may have produced unstable estimates.

## Author Contributions

**Melese Legesse Mitku:** conceptualization, investigation, methodology, validation, software, data curation, writing – original draft, writing – review and editing, formal analysis. **Yeniewa Kerie Anagaw:** conceptualization, investigation, funding acquisition, methodology, validation, visualization, writing – review and editing, software, formal analysis, project administration, data curation, resources, writing – original draft. **Abera Dessie Dagnaw:** investigation, validation, methodology, software, data curation. **Derso Teju Geremew:** conceptualization, investigation, validation, data curation, resources. **Yeshiwas Ayale Ferede:** investigation, writing – review and editing, methodology, software, data curation, validation. **Tewodros Denekew Haile:** conceptualization, investigation, validation, methodology, visualization, data curation. **Minichil Chanie Worku:** conceptualization, investigation, methodology, validation, software, data curation, resources. **Liknaw Workie Limenh:** conceptualization, investigation, validation, methodology, formal analysis, data curation. **Azmeraw Bekele:** investigation, visualization, validation, methodology, software, data curation. **Wondim Ayenew:** investigation, funding acquisition, methodology, validation, visualization, project administration, formal analysis, software, supervision, data curation.

## Funding

The authors have nothing to report.

## Ethics Statement

Ethical approval was obtained from the University of Gondar Institutional Review Board (Ref. No. SOP/116/2024). The data were collected after the study was approved by the institutional authority.

## Consent

Since the ages of all the study participants were above 18 years old, written informed consent was obtained from all study participants. The participants were informed of their rights before signing the informed consent, including the right to withdraw from the study at any time. To maintain the confidentiality of the participants, names and addresses were not recorded in the data collection forms. This study was performed in accordance with the Declaration of Helsinki.

## Conflicts of Interest

The authors declare no conflicts of interest.

## Transparency Statement

The lead author, Yeniewa Kerie Anagaw, affirms that this manuscript is an honest, accurate, and transparent account of the study being reported; that no important aspects of the study have been omitted; and that any discrepancies from the study as planned (and, if relevant, registered) have been explained.

## Data Availability

Data sharing not applicable to this article as no datasets were generated or analyzed during the current study.
